# Medical Opinion and Movement

**Published:** 1911-04-22

**Authors:** 


### Medical Opinion and Movement.

### Asphyxia of the New-born.

Dr. W. E. Fitch published a series of cases some months back, in which infants born in a state of asphyxia were resuscitated by means of aëration of the maternal surface of the placenta. Dr. H. Freund has just published a case in which the employment of this method was also completely successful. A young primipara, having been in labour for twenty-four hours, was admitted into the Sydenham Hospital. The pains were continuous, and two injections of scopomorphine were without effect, the uterus remaining in a state of veritable tetany. Two hours after admission, as the patient was becoming very feeble and the bruits of the fœtal heart were becoming weak, the forceps were applied under chloroform anæsthesia, and the child delivered in a state of asphyxia. The skin was livid, the heart beating visibly, the pulsations of the umbilical cord were perceptible on palpation, but there was no respiratory movement. The infant was placed in a basin of hot water and the placenta held in such a way that the maternal surface was exposed to the air, the surface being freed of blood clots with hot water. After a few minutes the child became a better colour, but there was no effort of respiration. For thirty-five minutes respiration continued by means of the maternal surface of the placenta exposed to the air. The pulsations of the cord were normal throughout this time, but they diminished in number and force as soon as the maternal surface of the placenta was placed on the table. On the child appearing again cyanosed, a current of oxygen was passed over the surface of the placenta, and this sufficed to restore the normal colour and improve the pulse. The cord was then divided, and the child immediately cried vigorously, and from that time things took a normal course.

### Adrenalin Inhalation.

The treatment of asthma by means of inhalations of powdered adrenalin has been referred to recently in these columns. Dr. Zülzer, of Berlin, has found a still more extended use for this treatment in cases of acute and chronic inflammation of the respiratory passages. He finds it especially efficacious in cases of acute laryngitis and acute bronchitis. In chronic bronchitis the treatment was not found to be so efficacious. In ordinary cases of pneumonia also the treatment did not appear to have any marked effect; but in certain cases of interstitial pneumonia complicated by a profuse catarrh two or three inhalations sufficed to cause a complete disappearance of these catarrhal symptoms. In two cases of pulmonary tuberculosis with moist râles localised at the apices of the lungs, these adrenalin inhalations similarly effected a complete removal of the catarrhal symptoms in a few days. The treatment does not produce any injurious effects. Sometimes the patients complain of a feeling of giddiness immediately after the inhalation, but this quickly passes off. The pulse and blood-pressure remain quite normal.

### Bilateral Empyema.

Dr. M. B. Fabrikant, of Kharkov, discusses the interesting question of bilateral empyema, its frequency, age incidence, prognosis, and treatment. According to statistics extending over 3,623 cases of pleuritic effusion, 155 were bilateral, showing a percentage of 4.3. Bilateral pleurisy, and especially double empyema, occurs chiefly in children. Of 85 cases of double empyema 59 were children under ten, 21 were between ten and twenty, and only five were over twenty years of age. In 118 cases collected by Dr. Fabrikant there were 74 cures and 44 deaths. But if one deducts from these 44 cases the deaths from pyæmia, the pre-operation deaths, and the deaths from accidental causes, etc., there remain only 11, or a mortality of 9.32 per cent. Whatever may be the significance of these figures, the chief point they emphasise is that double empyema is certainly curable, and by no means necessarily fatal. Thoracic surgery has so far improved of late that the fear of pneumothorax and its consequences is no longer a bar to operative interference in such cases. Double thoracotomy in cases of double empyema is no longer to be regarded as an operation of extreme severity and danger. In six recent cases in which double thoracotomy was practised five recovered completely, and the sixth only proved fatal on the fifty-second day. In only one case collapse occurred during the operation; but this did not prevent ultimate recovery. If the effusion is very large on both sides and the general infection severe, both sides should be opened freely at once, one after the other. On the other hand, if the condition is not so threatening, the author advises operation on one side first, by preference on the left, if the right side is not predominantly affected, and simple puncture and aspiration of the other, complete evacuation of this side being carried out a few days later. The intermediate stage between the evacuation of the two sides should not however be prolonged more than a few days, the whole object of treatment being to rid the chest of the purulent collections in as short a time as possible.

### Salicylate Injections.

Dr. Bouchard instituted some years ago injections of sodium salicylate solution directly into the affected part in cases of localised rheumatic affections. Dr. A. Seibert, of St. Francis' Hospital, New York, has had recourse to subcutaneous injections of sodium salicylate in much larger doses and as a method of general treatment, irrespective of the local rheumatic lesions. He reports several cases in which a rheumatic arthritis, affecting several joints, and persisting, in spite of large doses of salicylate administered by the mouth for two months or more, readily yielded to subcutaneous injections of sodium salicylate. He injects 10 to 15 cubic centimetres of a 20-per-cent. solution of sodium salicylate (equal to 30 to 45 grains) twice a day. Similarly successful results were obtained by the same treatment in three severe cases of rheumatic chorea. The involuntary movements ceased after the fourth injection, and all morbid symptoms disappeared, including fever, and in one case albuminuria, after the twelfth injection. The injections have the disadvantage of being extremely painful. In order to overcome this difficulty, Dr. Seibert first injects $\frac{1}{8}$ grain of cocaine in 30 minims of water after sterilising the skin with iodine, and a quarter of an hour later injects the sodium salicylate solution. In chronic cases the author uses a solution of 10 grammes of salicylic acid in 80 grammes of oil of sesame with 5 grammes of alcohol (to assist solution) and an equal quantity of camphor. The mixture must be sterilised before the addition of alcohol to avoid evaporation and precipitation of the salicylic acid. This solution appears to be more efficacious in chronic cases because it is less easily absorbed and eliminated, and therefore more lasting in its effect. The addition of camphor is especially indicated in the presence of rheumatic cardiac affections—endocarditis or pericarditis. The aqueous solution of sodium salicylate should be boiled immediately before its use. The oily solution of salicylic acid remains sterile after the first boiling.

### The Diagnosis of Scarlet Fever.

Rumpel, in the *Münchener Med. Woch.*, describes a new sign of scarlet fever. As long ago as 1907 Hecht had remarked that certain forms of cutaneous hæmorrhage were characteristic of the disease. He took a fold of skin either of the back or chest and pressed it hard for a few seconds between his thumb and index finger, after which petechial hæmorrhages appeared, easily in cases of scarlet fever, but with difficulty in other cases. The present observer makes use of this fact in producing the new sign, but instead of employing his thumb and index finger uses a rubber tourniquet. Characteristic cutaneous hæmorrhages appear below the ligature in cases of scarlet fever, and they may be small or extensive and petechial or very diffuse. The hæmorrhages which practically never appear in other diseases as the result of such pressure can be explained by supposing that the toxins of scarlet fever render the capillaries particularly vulnerable. In measles this weakness is much less common, and though it may occur the hæmorrhages are much less pronounced.

### The Renewal of Prescriptions.

M. Lacroix has proposed the following resolutions to a meeting of the Paris Therapeutical Society: (1) As regards physicians, when one of them prescribes a drug capable of giving rise to toxic symptoms, either as the result of an error in the subsequent use of the drug, or of an abuse which might be made of it, the prescription should bear, according to the text of the law, the amount of the toxic substance ordered, its mode of administration, and, when it seems to the physician necessary, the maximum number of times the prescription can be renewed without being countersigned again by him. (2) Every time that a pharmacist makes up a prescription in the cases alluded to by the laws and regulations relative to the sale of toxic substances in whatever form they may be, the aforesaid pharmacist must apply his seal to the prescription and give it a new number, even if it has already been inscribed in his register. (3) Solutions for hypodermic injection should in no case be supplied a second time without the special authorisation of the physician who ordered them in the first place. These proposals are worthy of consideration, not only in France, but in every country where self-drugging is prevalent. Their adoption would put an end to prescriptions being made family "heirlooms" and used in the treatment of ailments entirely foreign to the one for which they were originally prescribed.

### Lingual Goitres.

Under the heading "Solid thyroid tumours of the base of the tongue," Drs. E. Gellé and P. Bertein contribute an interesting article to *L'Echo Médicale du Nord.* They state that the canal of the thyroglossus, or the cord which replaces it, is often, as the result of anomalous development, the seat of pathological changes. Among these the most common are cystic tumours of the thyroid region, but solid neoplasms are met with also along the whole length of the tract from the foramen cæcum to the isthmus of the thyroid. The authors have themselves seen two cases of this last kind. The first was in a girl of eighteen, who complained of bleeding from the tongue when eating her breakfast the day before coming under observation, which bleeding, after stopping spontaneously, recurred twice again that day, and once the morning of the consultation. A central elastic tumour of the posterior portion of the tongue was found, richly supplied with veins, bleeding easily, and of the size of a nut. The left half of the tumour was larger than the right. Palpation was painless. The thyroid in the neck was of normal proportions. The tumour was removed under anæsthesia, and microscopic examination showed on the left side the structures of a thyroid gland, with here and there patches of epithelial tissue as are found in parenchymatous goitres. In the other half of the tumour thyroid tissue was difficult to find. It consisted for the most part in an abundant proliferation of glandular tissue, with a tendency in places towards cyst formation. The second case was that of a woman of forty, who came under treatment for respiratory difficulties which were found to be the result of a tumour the size of a tangerine orange situated at the base of the tongue, and which almost completely filled the pharynx. Deglutition was not much interfered with. Palpation revealed a firm elastic mass. Before an operation could be performed for the removal of the tumour the patient's condition necessitated a tracheotomy. Later the mass was removed by a median pharyngotomy, and the patient eventually was discharged cured. Histological examination revealed structures similar to those found in the first case. Clinically lingual goitres are found at all ages, but usually about puberty and especially in women.

